# Addressing frailty in older adults: an integrated challenge for health, science, and society

**DOI:** 10.18632/aging.206162

**Published:** 2024-11-27

**Authors:** Samuel Fernández-Carnero, Oliver Martínez-Pozas, Juan Nicolás Cuenca-Zaldívar, Eleuterio A. Sánchez-Romero

**Affiliations:** 1 Universidad de Alcalá, Facultad de Enfermería y Fisioterapia, Departamento de Fisioterapia, Grupo de Investigación en Fisioterapia y Dolor, Alcalá de Henares 28801, Spain; 2 Interdisciplinary Research Group on Musculoskeletal Disorders, Faculty of Sport Sciences, Universidad Europea de Madrid, Villaviciosa de Odón 28670, Spain; 3 Occupational Therapy, Rehabilitation and Physical Medicine, Escuela Internacional de Doctorado, Universidad Rey Juan Carlos, Alcorcón 28933, Spain; 4 Physiotherapy and Orofacial Pain Working Group, Sociedad Española de Disfunción Craneomandibular y Dolor Orofacial (SEDCYDO), Madrid 28009, Spain; 5 Research Group in Nursing and Health Care, Puerta de Hierro Health Research Institute-Segovia de Arana (IDIPHISA), Majadahonda 28222, Spain; 6 Physical Therapy Unit, Primary Health Care Center “El Abajón”, Las Rozas de Madrid 28231, Spain; 7 Universidad Europea de Madrid, Department of Physiotherapy, Faculty of Medicine, Health and Sports, Villaviciosa de Odón 28670, Spain

**Keywords:** frailty, health policy, dependency burden, ultrasound, sarcopenia

## Abstract

Introduction: The global shift towards an aging population presents significant challenges, particularly concerning frailty among older adults. Frailty, characterized by diminished strength and resilience, increases vulnerability to diseases and hospitalization.

Health Issues: Traditional diagnostic tools for frailty are costly and involve radiation risks, necessitating non-invasive, cost-effective methods like ultrasound. Frail older adults require intensive medical care, increasing healthcare costs and burdening systems.

Scientific Challenges: Research must adopt a multidimensional approach, considering physical, psychological, and social factors of frailty. There is a pressing need to develop accessible diagnostic tools and translate research findings into clinical practice. Integrating ultrasound with machine learning enhances diagnostic accuracy and predictive capabilities, facilitating personalized care.

Social Implications: Frailty reduces the quality of life for older adults, places emotional and financial burdens on families, and exacerbates health inequalities. It also leads to social isolation, diminishing the participation of older adults in community activities.

Future Directions: An integrated response involving public health policies, technological innovations, and education is necessary. Developing community health programs, implementing continuous health monitoring tools, and conducting awareness campaigns can significantly improve frailty management.

Conclusions: Tackling frailty is not only a health imperative but also a social and ethical responsibility. By addressing the intertwined health, scientific, and social challenges of frailty, we can ensure healthy and equitable aging for all, reflecting our commitment to improving the quality of life for older adults.

## INTRODUCTION

The global demographic shift towards an aging population is one of the most significant changes in the 21st century [1]. It is projected that 22% of the world’s population will be aged 60 years or older by 2050. This shift presents significant challenges for healthcare systems, communities, and society as a whole [2]. Frailty among older adults is a central issue in this context, with profound implications for health, scientific research, and social structures [3].

## Health challenges

Frailty is characterized by a decline in strength, endurance, and physiological function, which increases vulnerability to diseases, hospitalization, and mortality. Addressing frailty is a critical healthcare challenge.

1. **Complex Diagnosis:** Diagnosing frailty is often complex because it does not always manifest clearly and can be mistaken for normal aging. Traditional diagnostic tools, such as dual-energy X-ray absorptiometry (DXA) and computed tomography (CT), are costly and involve radiation risks, limiting their routine use in clinical practice. There is a pressing need for noninvasive, cost-effective, and accurate diagnostic methods to identify frailty early and efficiently [4].
2. **Healthcare System Burden:** Frail older adults require intensive and multidisciplinary medical care, placing heavy demand on healthcare resources. Frequent and prolonged hospitalizations due to frailty-related complications significantly increase healthcare costs [5]. This situation calls for a robust system capable of managing and supporting the complex needs of frail older adults, reducing hospital readmissions, and enhancing quality of care.
3. **Need for Personalized Interventions:** Treatments and rehabilitation programs must be tailored to the specific needs of each patient, considering their level of frailty and coexisting conditions [6, 7]. However, the lack of effective tools to assess and monitor frailty complicates the implementation of personalized care plans. Innovative approaches are necessary to develop and deploy interventions that can improve the health and well-being of frail individuals [8, 9].

## The role of ultrasound in frailty

Ultrasound technology represents a promising advancement in frailty diagnosis and management. Its use in evaluating muscle health provides critical insights that are both noninvasive and cost-effective, addressing several of the current limitations in frailty assessment [10].

1. **Non-Invasive and Accessible:** Unlike DXA and CT, ultrasound does not involve radiation, making it safer for repeated use, especially in older adults. It is also more accessible and cost-effective, facilitating broader implementation in clinical and community settings.
2. **Detailed Muscle Assessment:** Ultrasound allows the measurement of muscle thickness, cross-sectional area, and quality, which are crucial indicators of muscle health and frailty. These measurements can help to identify sarcopenia (loss of muscle mass and strength) early, which is a key component of frailty. Healthcare providers can better understand the progression of frailty by evaluating the muscle architecture and detecting changes over time.
3. **Real-Time Monitoring:** Ultrasound provides real-time imaging, enabling immediate assessment and monitoring. This capability is particularly beneficial in tracking the effectiveness of interventions aimed at improving muscle health and reducing frailty. Regular ultrasound evaluations can guide personalized treatment plans, ensuring that they are responsive to the patient’s current condition [10, 11].
4. **Integration with Machine Learning:** The Integration of ultrasound data with machine learning algorithms enhances the diagnostic accuracy and predictive power of frailty. Machine learning can analyze complex patterns in ultrasound images and correlate them with clinical outcomes, aiding the development of sophisticated predictive models. These models can help identify individuals at a higher risk of frailty and tailor interventions accordingly [12].

## Scientific challenges

Research on frailty faces several scientific challenges that need to be addressed to advance the understanding and management of this condition.

1. **Multidimensional Understanding:** Frailty is a multifaceted syndrome involving physical, psychological, and social aspects. Further research must integrate these dimensions to develop a holistic understanding of frailty. This requires interdisciplinary collaboration and comprehensive studies that examine how various factors contribute to the onset and progression of frailty [12].
2. **Development of Diagnostic Tools:** There urgent need to develop non-invasive, accessible, and precise diagnostic tools. Technologies such as ultrasound and machine learning algorithms show great potential in this area. Ultrasound, for example, offers a noninvasive and cost-effective way to measure muscle thickness and quality, which are critical indicators of frailty [12–15]. Machine learning can analyze large datasets to identify patterns and correlations, aiding the development of predictive models for frailty [12].
3. **Translational Research:** Research findings must be effectively translated into clinical practice. This requires collaboration among scientists, healthcare providers, and policymakers to implement new diagnostic and treatment strategies [14, 15]. Translational research bridges the gap between laboratory discoveries and real-world applications, ensuring that advances in understanding frailty lead to tangible benefits for patients [16].

## Social implications

The impact of frailty extends beyond health and scientific research and affects the social fabric in several ways.

1. **Quality of Life:** Frailty significantly reduces the quality of life of older adults, limiting their ability to perform daily activities and enjoy an autonomous and dignified life. Addressing frailty is crucial for improving the overall well-being and life satisfaction of the elderly population [17].
2. **Family Burden:** Families of frail individuals often face substantial emotional and financial burden. Caregivers may experience stress, burnout, and economic hardships. Providing adequate support for caregivers is essential to alleviate their burden and ensure that they can provide effective care for their loved ones [18].
3. **Health Inequalities:** Frailty can exacerbate health inequalities because vulnerable populations with fewer resources have less access to appropriate diagnostics and treatments. Addressing these disparities is crucial for ensuring equitable healthcare for all older adults, regardless of their socioeconomic status [19].
4. **Social Participation:** The integration and active participation of older adults in society is vital. Frailty can lead to social isolation, reducing interactions, and contributions to the community. Promoting social inclusion and participation of the elderly is essential for enhancing their quality of life and societal engagement [20].

### Need for an integrated response

Addressing frailty in older adults requires an integrated response involving multiple stakeholders and approaches:

1. **Public Health Policies:** Developing and implementing public health policies that promote prevention, early detection, and effective management of frailty are essential. Investing in community health programs that support older adults and their families can significantly impact the management of frailty [5, 12].
2. **Technological Innovation:** Fostering research and the development of new diagnostic and therapeutic technologies is crucial. Implementing digital tools to continuously monitor and manage the health of older adults in a personalized manner can lead to better outcomes and quality of care [12, 21].
3. **Education and Awareness:** Raising awareness of frailty and the importance of healthy aging through educational campaigns is vital. Training and supporting healthcare professionals to improve frailty management in clinical practice can enhance the care provided to frail older adults [22].

## CONCLUSIONS

Frailty in older adults is a complex issue that requires a multi-dimensional approach. Intertwined health, scientific, and social challenges require innovative, collaborative solutions. By addressing frailty effectively, we can improve the quality of life of older adults, ensure healthy and equitable aging, and reduce the burden on families and healthcare systems. Tackling frailty is not just a health imperative, but also a social and ethical one that reflects our values and priorities as a society.
